# Flap fixation in preventing seroma formation after mastectomy: an updated meta-analysis

**DOI:** 10.1007/s13304-021-01049-9

**Published:** 2021-04-22

**Authors:** Nunzio Velotti, Gennaro Limite, Antonio Vitiello, Giovanna Berardi, Mario Musella

**Affiliations:** Department of Advanced Biomedical Sciences, University of Naples “Federico II”, Via Pansini n.5, 80131 Naples, Italy

**Keywords:** Mastectomy, Flap fixation, Seroma, Flap quilting

## Abstract

Seroma formation following mastectomy is one of the most experienced complications, with a very variable incidence ranging from 3 to 90%. In recent years, many publications have been realized to define an effective technique to prevent its formation and several approaches have been proposed. Given the potential of flap fixation in reducing seroma formation, we performed a meta-analysis of the literature to investigate the role of this approach as definitive gold standard in mastectomy surgery. Inclusion criteria regarded all studies reporting on breast cancer patients undergoing mastectomy with or without axillary lymph node dissection; studies that compared mastectomy with flap fixation to mastectomy without flap fixation were selected. Papers were eligible for inclusion if outcome was described in terms of seroma formation. As secondary outcome, also surgical site infection (SSI) was evaluated. The included studies were 12, involving 1887 female patients: 221/986 (22.41%) patients experienced seroma formation after flap fixation and 393/901 (43.61%) patients had this complication not receiving flap fixation, with a significant statistical difference between the two groups (OR = 0.267, *p* = 0.001, 95% CI 0.153, 0.464). About, SSI 59/686 (8.6%) in flap fixation group and 67/686 (9.7%) in patients without flap fixation, with no statistical differences between groups (OR = 0.59, *p* = 0.056, 95% CI 0.344, 1.013).

The heterogeneity between included studies does not allow us to reach definitive conclusions but only to suggest the strong evaluation of this approach after mastectomy in seroma preventing and SSI reduction.

**Supplementary Information:**

The online version contains supplementary material available at 10.1007/s13304-021-01049-9.

## Introduction

Seroma formation following mastectomy is one of the most experienced complications, with a very variable incidence ranging from 3 to 90%. It is defined as a serous fluid collection that develops under the skin flaps during mastectomy or in the axillary dead space after axillary dissection [1]; some surgeons consider it to be an unavoidable surgical nuisance and, although it is not life threatening, it can lead to patient discomfort, repeated aspirations with the risk of infection, prolonged hospital stay, delayed wound healing and delay in commencing adjuvant therapies [2]. Current literature has already stated the biological mechanisms related to seroma formation and the associated risk factors: factors including the age of the patient, obesity, the extent of axillary lymph node involvement and the type and extent of breast surgery are the most cited responsible of this complication [3, 4] .

In recent years, many publications have been realized to define an effective technique to prevent seroma formation and all the authors agree that the best strategy is to reduce the dead space after mastectomy [5]; to achieve this result, several approaches have been proposed including closed-suction drainage, quilting of the skin flaps or application of adhesive tissue glue [6].

Given the potential of flap fixation in reducing seroma formation, we performed a meta-analysis of the literature to investigate the role of this approach as definitive gold standard in mastectomy surgery.

## Materials and methods

A protocol for this analysis was prospectively developed, with specific objectives, detailed criteria for study selection and evaluation of study quality, identification of the outcomes and of the statistical methods.

### Literature search strategy

To identify all available studies, a systematic search was performed according to PRISMA (Preferred Reporting Items for Systematic reviews and Meta-Analyses) flowchart in all electronic databases (PubMed, Web of Science, Scopus, and EMBASE). We used medical subject headings (MeSH) and free-text words using the following search terms in all possible combinations: flap fixation, flap quilting, mastectomy, and breast. The last search was performed in December 2020.

According to PICO framework (Problem/Population, Intervention, Comparison and Outcome), study selection criteria was exactly defined. The main outcome measure of this review was symptomatic seroma, defined as seroma requiring any form of surgical intervention; the secondary outcome was surgical site infection (SSI). The search strategy was limited to articles written in English language.

### Studies selection and data extraction

Inclusion criteria regarded all studies reporting on breast cancer patients undergoing mastectomy with or without axillary lymph node dissection; studies that compared mastectomy with flap fixation to mastectomy without flap fixation were selected. Papers were eligible for inclusion if outcome was described in terms of seroma formation.

Studies not written in English and papers regarding animal studies, such as studies involving patients undergoing direct breast reconstruction were excluded.

Two independent authors (VS, LV) analyzed each article and performed the data extraction independently. Duplicate studies were removed. Two other authors (NV, AV) further reviewed independently the eligibility of studies in abstract form and in full text by assessing if the inclusion criteria and outcome measures were met. In case of disagreement, a fourth investigator was consulted (MM). Discrepancies were resolved by consensus.

Data regarding sample size, age, Body Mass Index (BMI), smoking habit, neoadjuvant therapy, tumor stage, histological type, number of lymph node harvested, surgical site infection and seroma formation were obtained for each group (flap fixation vs no flap fixation) of all included studies.

### Statistical analysis

Dichotomous variables were pooled using the odds ratio (OR) with a 95% CI. In case of zero total events trials, we used the risk difference (RD) as effect measure to maintain analytic consistency and to incorporate all available data. The overall effect was tested using *Z* scores and significance was set at *p* < 0.05. Statistical analysis was realized with using Comprehensive Meta-analysis [Version 2, Biostat, Englewood NJ (2005)].

Heterogeneity was investigated by the use of *I*^2^ statistic. For *I*^2^ of between 0 and 30%, heterogeneity was considered as probably not important, between 30 and 60% moderate, between 50 and 90% substantial, and between 75 and 100% considerable [7].

To be as conservative as possible, the random effect method was used for all analyses to take into account the variability among included studies.

Furthermore, we performed a meta-regression analysis to assess the possible effect of oncological (tumor stage, histological type, and number of lymph node harvested) and demographic variables (age, BMI, smoking habit, and neoadjuvant therapy) on the incidence of seroma formation and SSI. To assess the possible effect of such variables in explaining different results observed across studies, we planned to perform meta-regression analyses after implementing a regression model with incidence of seroma and SSI as dependent variable (*y*) and the age, BMI, smoking habit, neoadjuvant therapy, tumor stage, histological type and number of lymph node harvested as independent variables (*x*).

### Risk of bias assessment

Publication bias was assessed by the Egger’s test and represented graphically by funnel plots for each outcome. Visual inspection of funnel plot asymmetry was performed to address for possible small-study effect, and Egger’s test was used to assess publication bias, over and above any subjective evaluation [8]. A *p* < 0.10 was considered statistically significant. In case of a significant publication bias, the Duval and Tweedie’s trim and fill method was used to allow for the estimation of an adjusted effect size [9].

### Quality assessment

The quality of each included study was assessed. For Randomized Clinical Trial (RCT), it was evaluated according to the Cochrane Collaboration tool for assessing risk of bias: seven distinct domains were identified and evaluated as ‘‘Low risk of bias’’ or ‘‘High risk of bias’’ or ‘‘Unclear’’: sequence generation, allocation concealment, blinding of participants, blinding of outcome assessment, incomplete outcome data, selective outcome reporting, and other potential threats to validity (Appendix 1a in ESM).

For non-randomized studies, the Newcastle–Ottawa Scale (NOS) was used [10]: the NOS contains eight items, categorized into three domains: (1) selection of study (four points); (2) comparability of groups (two points); (3) ascertainment of exposure and outcomes (three points) for case–control and cohort studies, respectively. A star system is used to allow a semi-quantitative assessment and researchers assign up to a maximum of nine points (Appendix 1b in ESM).

## Results

After excluding duplicate results, the search retrieved 56 articles. Of these studies, 32 were excluded because they were off the topic after scanning the title and/or the abstract, and 3 because they were reviews/comments/case reports. One study was excluded after full-length paper evaluation for lack of data, five studies were excluded for language and three studies because of no full text available. Thus, 12 studies were included in the analysis [11–22] (Appendix 2 in ESM).

### Studies characteristics

The included studies comparing flap fixation and no flap fixation approach to mastectomy were 12 [11–22], involving 1887 female patients, whereof 986 cases underwent flap fixation and 901 did not received flap fixation. We identified six retrospective studies [15–20], two prospective studies [12, 13] and four randomized controlled trials [11, 14, 21, 22].

Major characteristics of included studies are shown in Table 1. The mean age varied from 71 to 44 years and the mean BMI varied from 24 kg/m^2^ to 30.9 kg/m^2^. Smoking habit was reported in 8 studies [11, 13, 16–20, 22] for a total of 270 smoking patients. Tumor stage was reported only in 2 studies [12, 14] for a total of 190 patients and similarly histological type was reported only by 2 authors [12, 21] over 160 patients; about number of lymph node harvested, it was identified in 3 studies [14, 20, 21], varying from a mean of 7.6 ± 1.9–30 ± 16 nodes. Finally, 252 patients have had neoadjuvant therapy, reported in 8 included studies [11, 12, 14–16, 19, 20, 22].

**Table 1:** Data from included studies

Authors	Flap fixation	Patients, *n*	Age	BMI	Smoker, n	Neoadjuvant, n	Lymph node harvested	Seroma, n. (%)	SSI, *n*. (%)
Almond et al. 2010	Yes	214	46 ± 9	40.9 ± 1.5	5	–		37 (17.2)	–
	No	115	48 ± 9.6	40.2 ± 1.3	10	–		35 (30.4)	–
Britt Ten Walde et al. 2010	Yes	89	62 ± 14.4	26.35 ± 6.05	–	11		20 (2.2)	10 (11.2)
	No	87	60 ± 13.6	26.38 ± 4.38	–	7		70 (80.4)	27 (31)
de Rooij et al. 2020	Yes	214	65.3 ± 13.5	27.85 ± 5.2	41	40		–	–
	No	115	64.1 ± 12.6	27.4 ± 5.2	23	29		–	–
Eichler et al. 2016	Yes	32	67 ± 13	26 ± 7	0	4		8 (25)	–
	No	173	62 ± 14	27 ± 7	24	44		27 (15.6)	–
Granzier et al. 2016	Yes	126	65.2 ± 12.9	27.5 ± 4.8	27	27		11 (8.7)	14 (11.1)
	No	61	63.2 ± 12.5	27.1 ± 5.1	18	16		14 (22.9)	9 (14.7)
Khater et al. 2015	Yes	60	46 ± 7	30.5 ± 1.8	–	–	19 ± 3	–	–
	No	60	44 ± 8	30.9 ± 1.5	–	–	18 ± 3	–	–
Ouldamer et al. 2015	Yes	59	56.8 ± 11.9	25.6 ± 4.9	11	11	8 ± 3.45	–	5 (8.5)
	No	60	61 ± 14.5	25.1 ± 5	8	15	7.6 ± 1.9	–	2 (3.3)
Rajkumar Kottayasami et al. 2013	Yes	49	48 ± 10.5	24.8 ± 3.1	–	13	14 ± 6	4 (8.2)	–
	No	101	50 ± 10	25.4 ± 4.6	–	17	30 ± 16	17 (16.8)	–
Sakkary et al. 2012	Yes	20	51 ± 12.5	–	–	3		2 (10)	–
	No	20	54 ± 17	–	–	6		3 (15)	–
Van Bastelar et al. 2016	Yes	92	71 ± 11	–	21	–		33 (35.9)	11 (11.9)
	No	88	67 ± 13	–	21	–		52 (59.1)	14 (15.9)
Van Bastelar et al. 2017	Yes	142	64.5 ± 13	–	33	–		58 (40.8)	19 (13.6)
	No	64	69	–	24	–		52 (81.2)	15 (23.4)
Van Bastelar et al. 2019	Yes	27	69.55 ± 12.6	27.5 ± 5.1	4	5		–	–
	No	13	67.2 ± 12.2	29 ± 7.2	0	4		–	–

Data are expressed as mean ± standard deviation where not differently indicated

### Seroma

**Fig. 1:**
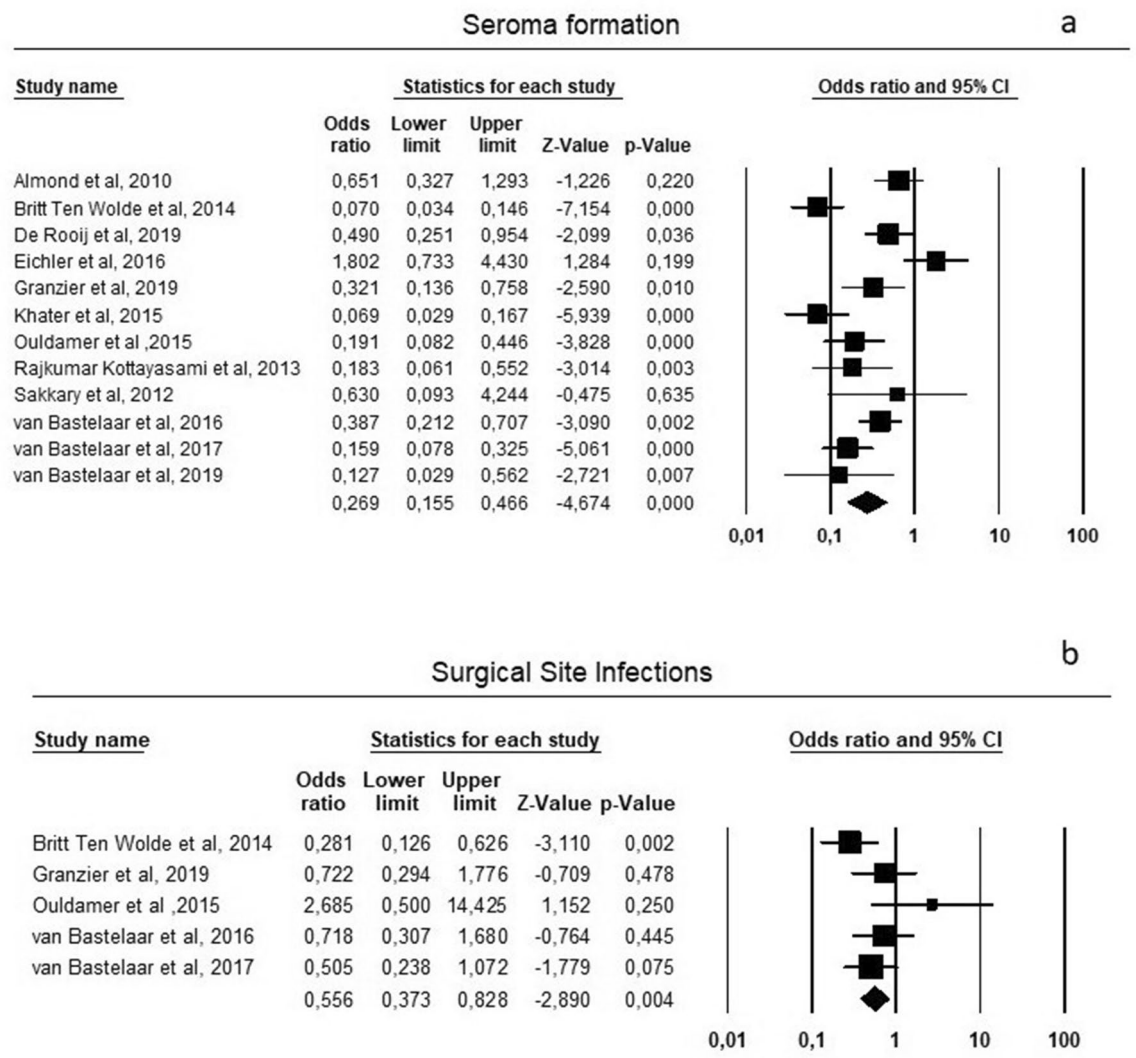
**a** Primary outcome: seroma formation; **b** secondary outcome: SSI

Results about seroma formation are shown in Fig. 1a. In details, seroma was reported by all the authors, involving 614/1887 patients (32.5%); 221/986 (22.41%) patients experienced seroma formation after flap fixation and 393/901 (43.61%) patients had this complication not receiving flap fixation, with a significant statistical difference between the two groups (OR = 0.267, *p* = 0.001, 95% CI 0.153, 0.464) and a significant heterogeneity among studies (*I*^2^ = 80.17%; *p* = 0.001).

### SSI

Secondary outcome of SSI was reported by 5 authors [11, 15, 17, 18, 20] involving 126/686 patients (18.3%), 59/686 (8.6%) in flap fixation group and 67/686 (9.7%) in patients without flap fixation, with no statistical differences between the groups (OR = 0.59, *p* = 0.056, 95% CI 0.344, 1.013) and no statistically significant heterogeneity among studies (*I*^2^ = 41.99%; *p* = 0.141) (Fig. 1b).

### Meta-regression

**Fig. 2:**
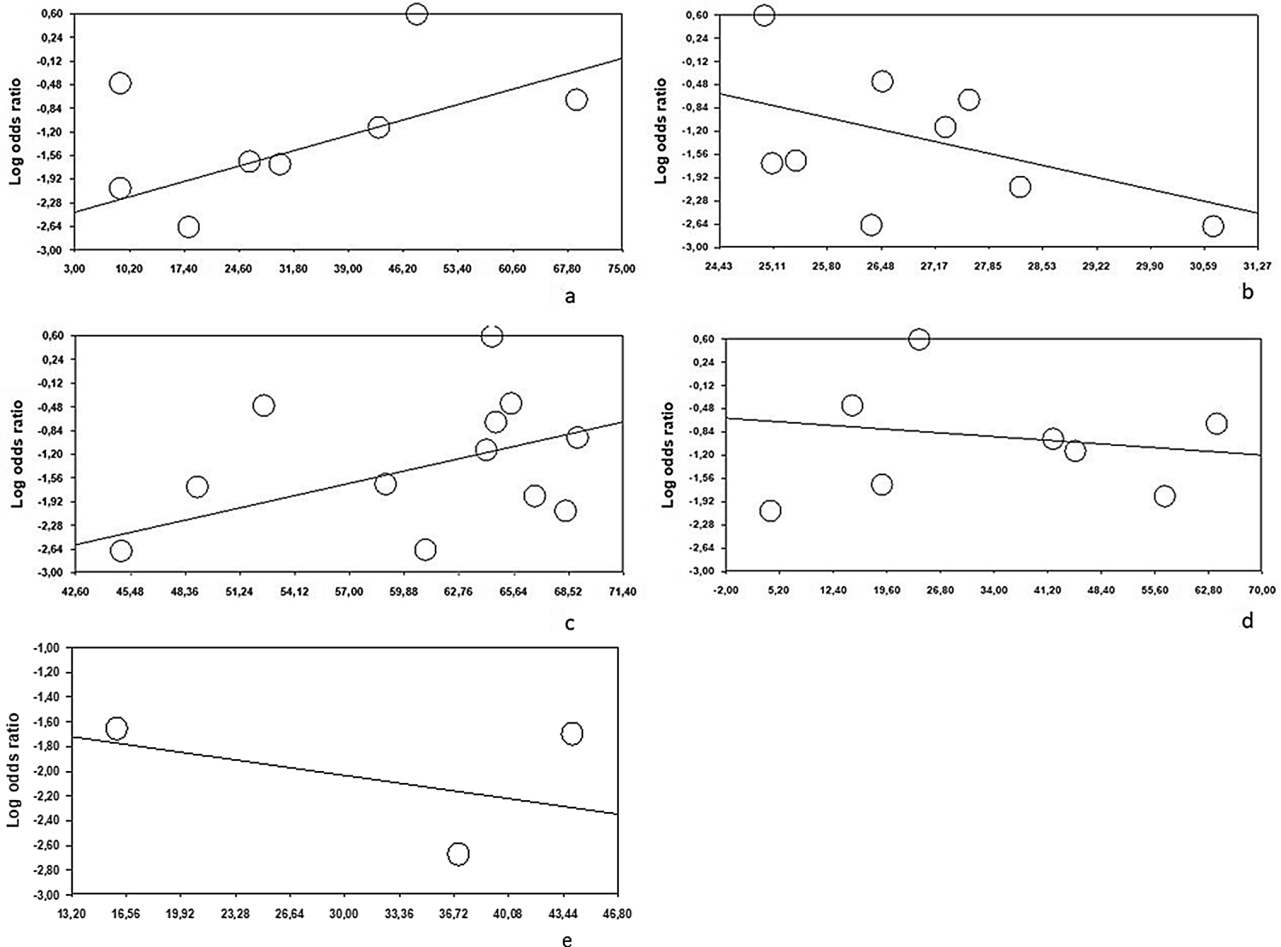
Meta-regression analysis over the primary outcome: **a** neoadjuvant therapy, **b** BMI, **c** age, **d** smoking habit, and **e** harvested lymph node

We found seroma formation was influenced by neoadjuvant therapy (*Z*-score 4.02, *p* = 0.001), BMI (*Z*-score − 2.98, *p* = 0.001) and patients’ age (*Z*-score 3.67, *p* = 0.001); on the other side, smoking habit (*Z*-score − 1.06, *p* = 0.28) and harvested lymph node (*Z*-score − 0.81, *p* = 0.41) do not impact seroma rate (Fig. 2a–e).

**Fig. 3:**
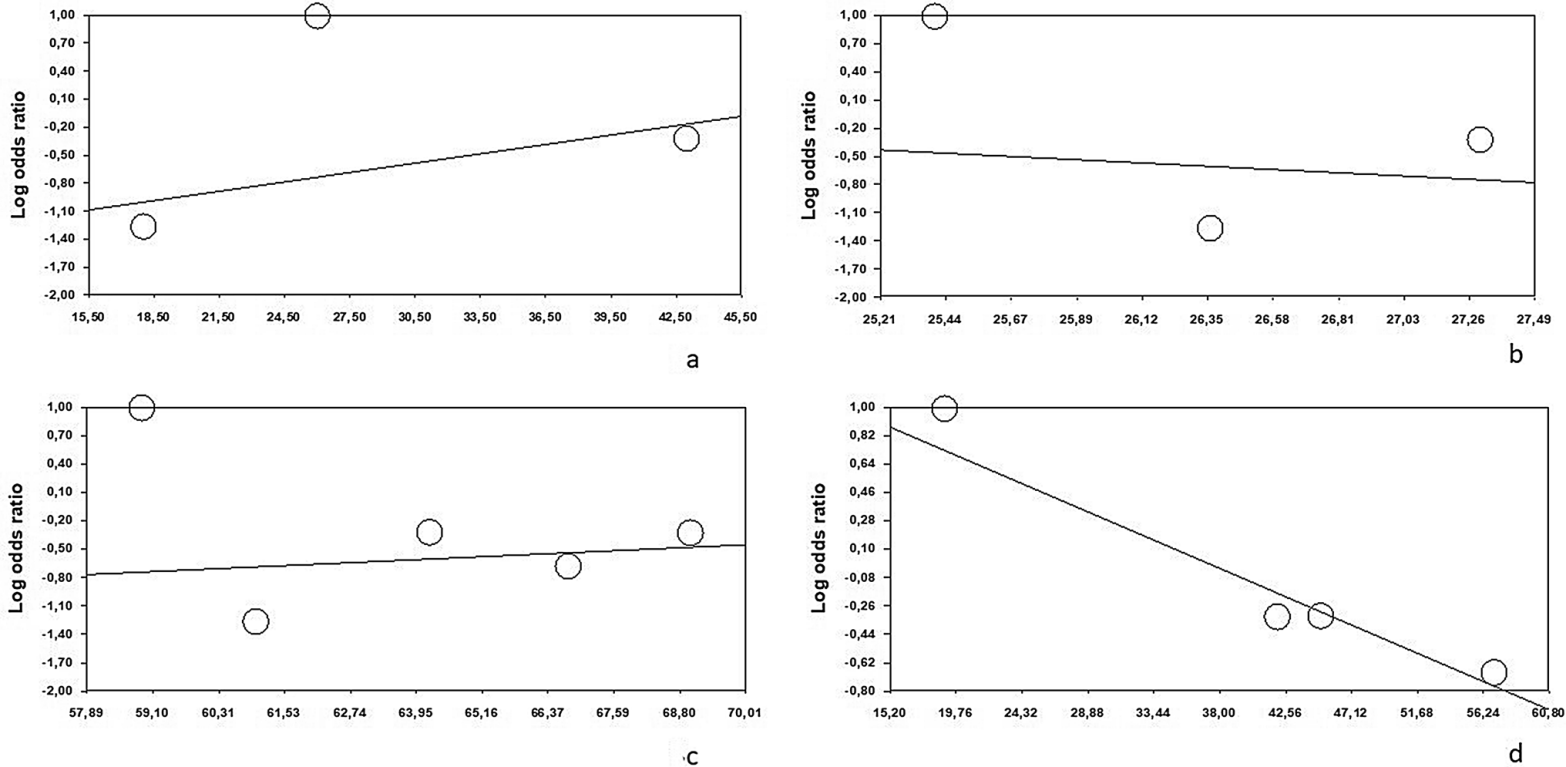
Meta-regression analysis over the secondary outcome: **a** neoadjuvant therapy, **b** BMI, **c** age, and **d** smoking habit

About SSI incidence, neoadjuvant therapy (*Z*-score 1.36, *p* = 0.17), BMI (*Z*-score − 0.32, *p* = 0.74), patients’ age (*Z*-score 0.41, *p* = 0.67) and smoking habit (*Z*-score 1.33, *p* = 0.18) do not impact complication rate (Fig. 3a–d).

### Publication bias

**Fig. 4:**
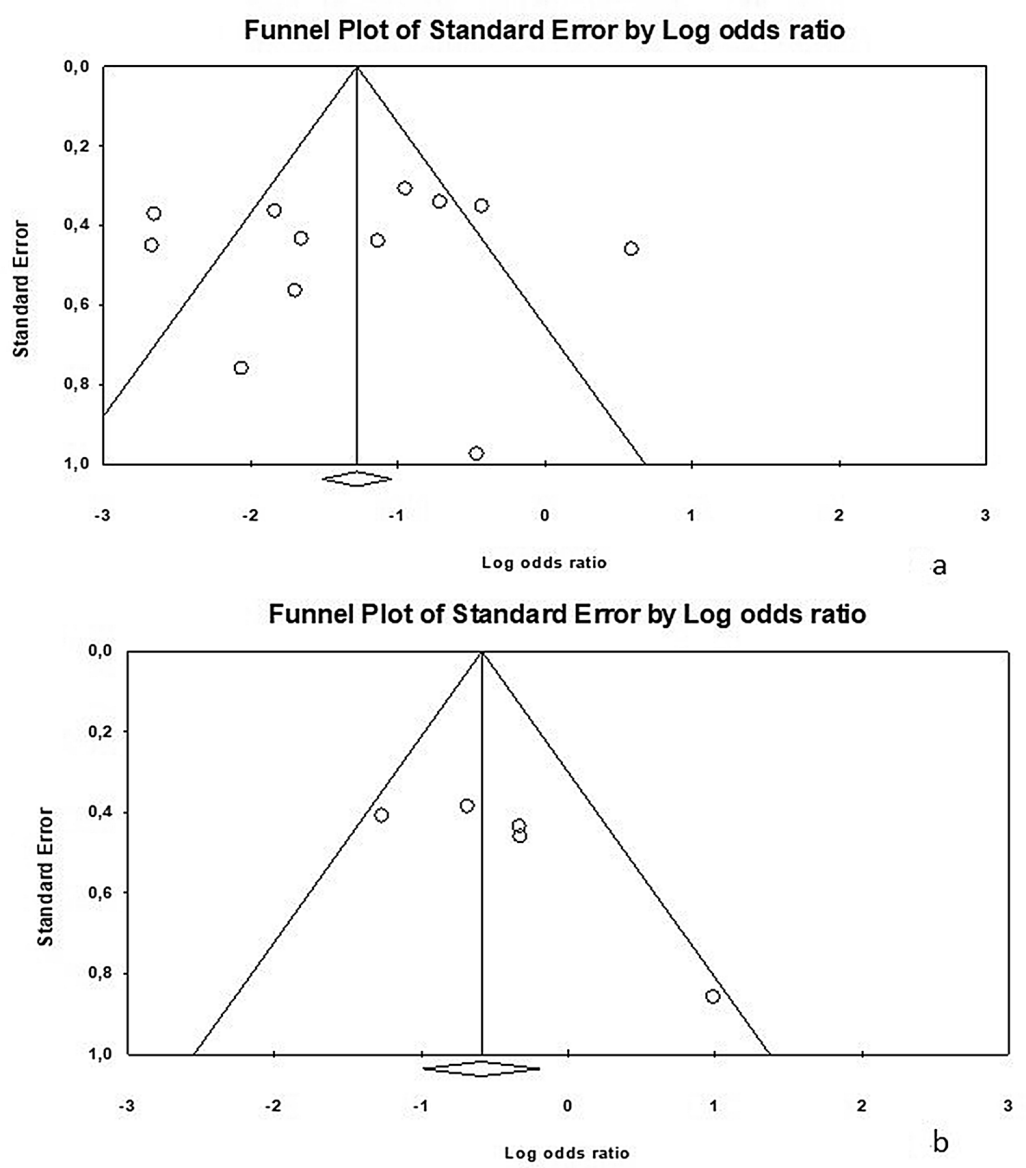
Publication bias: **a** seroma formation and **b** SSI

Since it is recognized that publication bias can affect the results of meta-analyses, we attempted to assess this potential bias using funnel plot analysis. The distribution of studies evaluating seroma formation (*p* = 0.76) and SSI (*p* = 0.10) was symmetrical and no publication bias was found by the Egger’s test. Results of risk of bias assessment are reported in Fig. 4a, b.

## Discussion

Modern screening programs have drastically reduced the mastectomy approach in favor of a more conservative surgery; despite that diagnostic and operational technological advances, preventing seroma formation and its complications after mastectomy remains challenging and considerable research has been done concerning the pathophysiology [6, 23]. By this point of view, the formation and its bothersome sequelae are of clinical importance both for patient discomfort, the possibility of infections and delayed wound healing even to surgical reinterventions [24].

In the past decades, many authors identified the application of suction drainage as the only effective solution to the problem of seroma. More recently, research has focused on the approach of closing the dead space and the studies favoring flap fixation after mastectomy has shown a substantial gain; for this reason, a growing number of studies have been published evaluating the effect of this approach on seroma formation following mastectomy or modified radical mastectomy [11–22].

Particularly, Rajkumar Kottayasamy et al., [14] in the first RCT on this topic, found that the obliteration of the dead space after breast cancer surgery by suture flap fixation is a safe and easy procedure, which significantly reduces postoperative seroma formation and duration of drainage. In addition, Khater, in a randomized controlled study carried out among 120 females who were candidates for mastectomy and axillary clearance, stated that quilting of the mastectomy flaps caused a significantly lower incidence of seroma and a shorter duration till seroma resolution with a smaller volume of drainage [21].

The recent SAM trial of Granzier and colleagues [11] on a total of 187 patients demonstrated that flap fixation with either sutures or adhesive tissue glue reduces the number of seroma aspirations when compared to simple wound closure. Same results were reported in the 2020 SARA trial from de Rooji et al. [22] which stated, on 250 patients, that flap fixation omitting closed-suction drainage in mastectomy is an effective approach to reduce seroma formation.

Despite substantial heterogeneity among the 12 studies included in our analysis, the evidence that flap fixation could reduce seroma formation seems convincing. Our results underlined a significant reduction in seroma formation when patients underwent flap fixation (22.41% vs 43.61%, OR = 0.267, *p* = 0.001). The meta-regression analysis suggested this primary outcome was influenced by BMI and patients’ age as stated in the previous literature [25], but, for the first time, we found an impact also of neoadjuvant therapy. About this last evidence, an explanation could be found in the study of van Bastelaar on interleukin-6 (IL-6) in seroma of patients undergoing mastectomy: the authors found that high levels of IL-6 are associated with clinical seroma formation 3 months after surgery [19]. Moreover, in a recent study on patients receiving neoadjuvant chemotherapy for locally advanced breast cancer, serum IL-6 levels were significantly higher before, during and after termination of chemotherapy [26]. Considering these data, our result may be related to the rise in this biomarker as possible responsible of increased seroma formation in patients who underwent neoadjuvant therapy.

We found also a reduction from 9.7 to 8.6% of SSI when flap fixation technique is applied. Britt ten Wolde [15] first described, in a study on 176 patients who underwent mastectomy and axillary lymph node dissection, a reduction of SSIs from 31.0 to 11.2% in patients receiving fixation of the skin flaps to the underlying muscles. In addition, Ouldamer et al. [20] found a quilting approach to the skin flap after mastectomy leads to a reduction in surgical site infections when compared with a conventional wound closure (2.3% vs. 11.6%). Of interest, none of the factors considered in the meta-regression analysis affects this outcome.

## Conclusions

In conclusion, the oncological radicality of mastectomy cannot ignore the objective of reducing its most frequent complication; by this point of view, the flap fixation approach has shown a good safety profile in terms of reduction of both seroma formation and SSI. Furthermore, particular attention should be paid to neoadjuvant therapy which has shown a direct impact on the main outcome of our analysis.

The major limitation of our study is represented by the heterogeneity between the selected studies, probably also due to the subjective method of reporting seroma formation and if no objective criteria are set for reporting seroma, a great variation in its incidence may result; this does not allow us to reach definitive conclusions but only to suggest the strong evaluation of this approach after mastectomy. Further studies will be needed to define a real gold standard and investigate the best methodology to obtaining an effective fixation of the flap.

## Supplementary Information

Below is the link to the electronic supplementary material.

Supplementary file1 (JPG 40 KB) Appendix 1a: Cochrane Collaboration tool for assessing risk of bias

Supplementary file2 (DOCX 14 KB) Appendix 1b: NOS Quality assessment

Supplementary file3 (DOCX 18 KB) Appendix 2: PRISMA flowchart

## References

[1] Pogson CJ, Adwani A, Ebbs SR (2003). Seroma following breast cancer surgery. Eur J Surg Oncol.

[2] Unalp HR, Onal MA (2007). Analysis of risk factors affecting the development of seromas following breast cancer surgeries: seromas following breast cancer surgeries. Breast J.

[3] Gonzalez EA, Saltzstein EC, Riedner CS, Nelson BK (2003). Seroma formation following breast cancer surgery. Breast J.

[4] Hashemi E, Kaviani A, Najafi M, Ebrahimi M, Hooshmand H, Montazeri A (2004). Seroma formation after surgery for breast cancer. World J Surg Oncol.

[5] van Bemmel AJ, van de Velde CJ, Schmitz RF, Liefers GJ (2011). Prevention of seroma formation after axillary dissection in breast cancer: a systematic review. Eur J Surg Oncol.

[6] Agrawal A, Ayantunde AA, Cheung KL (2006). Concepts of seroma formation and prevention in breast cancer surgery. ANZ J Surg.

[7] Higgins JP, Thompson SG, Deeks JJ, Altman DG (2003). Measuring inconsistency in meta-analyses. BMJ.

[8] Sterne JA, Egger M, Smith GD (2001). Systematic reviews in health care: Investigating and dealing with publication and other biases in meta-analysis. BMJ.

[9] Duval S, Tweedie R (2000). Trim and fill: a simple funnel-plot-based method of testing and adjusting for publication bias in meta-analysis. Biometrics.

[10] Stang A (2010). Critical evaluation of the Newcastle-Ottawa scale for the assessment of the quality of nonrandomized studies in meta-analyses. Eur J Epidemiol.

[11] Granzier RWY, van Bastelaar J, van Kuijk SMJ, Hintzen KFH, Heymans C, Theunissen LLB, van Haaren ERM, Janssen A, Beets GL, Vissers YLJ (2019). Reducing seroma formation and its sequelae after mastectomy by closure of the dead space: the interim analysis of a multi-center, double-blind randomized controlled trial (SAM trial). Breast.

[12] Sakkary MA (2012). The value of mastectomy flap fixation in reducing fluid drainage and seroma formation in breast cancer patients. World J Surg Oncol.

[13] Almond LM, Khodaverdi L, Kumar B, Coveney EC (2010). Flap anchoring following primary breast cancer surgery facilitates early hospital discharge and reduces costs. Breast Care (Basel).

[14] Kottayasamy Seenivasagam R, Gupta V, Singh G (2013). Prevention of seroma formation after axillary dissection–a comparative randomized clinical trial of three methods. Breast J.

[15] ten Wolde B, van den Wildenberg FJ, Keemers-Gels ME, Polat F, Strobbe LJ (2014). Quilting prevents seroma formation following breast cancer surgery: closing the dead space by quilting prevents seroma following axillary lymph node dissection and mastectomy. Ann Surg Oncol.

[16] Eichler C, Fischer P, Sauerwald A, Dahdouh F, Warm M (2016). Flap adhesion and effect on postoperative complication rates using Tissuglu® in mastectomy patients. Breast Cancer.

[17] van Bastelaar J, Beckers A, Snoeijs M, Beets G, Vissers Y (2016). Flap fixation reduces seroma in patients undergoing mastectomy: a significant implication for clinical practice. World J Surg Oncol.

[18] van Bastelaar J, Theunissen LLB, Snoeijs MGJ, Beets GL, Vissers YLJ (2017). Flap fixation using tissue glue or sutures appears to reduce seroma aspiration after mastectomy for breast cancer. Clin Breast Cancer.

[19] van Bastelaar J, Granzier R, van Roozendaal LM, van Kuijk SMJ, Lerut AV, Beets G, Hadfoune M, Olde Damink S, Vissers YLJ (2019). Analysis of TNF-α and interleukin-6 in seroma of patients undergoing mastectomy with or without flap fixation: is there a predictive value for seroma formation and its sequelae?. Surg Oncol.

[20] Ouldamer L, Caille A, Giraudeau B, Body G (2015). Quilting suture of mastectomy dead space compared with conventional closure with drain. Ann Surg Oncol.

[21] Khater A, Elnahas W, Roshdy S, Farouk O, Senbel A, Fathi A, Hamed E, Abdelkhalek M, Ghazy H (2015). Evaluation of the quilting technique for reduction of postmastectomy seroma: a randomized controlled study. Int J Breast Cancer.

[22] de Rooij L, van Kuijk SMJ, Granzier RWY, Hintzen KFH, Heymans C, Theunissen LLB, von Meyenfeldt EM, van Essen JA, van Haaren ERM, Janssen A, Vissers YLJ, Beets GL, van Bastelaar J (2020). Reducing seroma formation and its sequelae after mastectomy by closure of the dead space: a multi-center, double-blind randomized controlled trial (SAM-Trial). Ann Surg Oncol.

[23] Kumar S, Lal B, Misra MC (1995). Post-mastectomy seroma: a new look into the aetiology of an old problem. J R Coll Surg Edinb.

[24] Carless PA, Henry DA (2006). Systematic review and meta-analysis of the use of fibrin sealant to prevent seroma formation after breast cancer surgery. Br J Surg.

[25] van Bastelaar J, van Roozendaal L, Granzier R, Beets G, Vissers Y (2018). A systematic review of flap fixation techniques in reducing seroma formation and its sequelae after mastectomy. Breast Cancer Res Treat.

[26] Paz MFCJ, Gomes Júnior AL, Islam MT, Tabrez S, Jabir NR, Alam MZ, Machado KC, de Alencar MVOB, Machado KC, Ali ES, Mishra SK, Gomes LF, Sobral ALP, e Sousa JMC, de Souza GF, Melo-Cavalcante AAC, da Silva J (2018). Assessment of chemotherapy on various biochemical markers in breast cancer patients. J Cell Biochem..

