# Supplementary figures and images for: Flap fixation in preventing seroma formation after mastectomy: an updated meta-analysis
Source: Updates Surg. 2021 Apr 22;73(4):1307–14. doi: 10.1007/s13304-021-01049-9 (PMC8397649; doi:10.1007/s13304-021-01049-9)

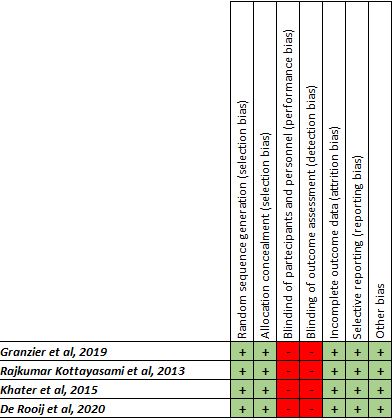

Supplement: Supplementary file 1 — Supplementary file1 (JPG 40 KB) Appendix 1a: Cochrane Collaboration tool for assessing risk of bias [file 13304_2021_1049_MOESM1_ESM.jpg]
