# Supplementary material for: Flap fixation in preventing seroma formation after mastectomy: an updated meta-analysis
Source: Updates Surg. 2021 Apr 22;73(4):1307–14. doi: 10.1007/s13304-021-01049-9 (PMC8397649; doi:10.1007/s13304-021-01049-9)
Supplement: Supplementary file 2 — Supplementary file2 (DOCX 14 KB) Appendix 1b: NOS Quality assessment [file 13304_2021_1049_MOESM2_ESM.docx]

| **References** | **Selection** | | | | **Comparability** | | **Outcome assessment** | | |
| --- | --- | --- | --- | --- | --- | --- | --- | --- | --- |
|  |  | | | |  | |  | | |
|  | **1** | **2** | **3** | **4** | **1** |  | **1** | **2** | **3** |
| Sakkary et al 2012 | ***** | ***** | ***** | ***** | ****** |  | ***** | ***** |  |
| Almond et al 2010 | ***** | ***** | ***** | ***** | ****** |  | ***** | ***** |  |
| ten Wolde et al 2014 | ***** | ***** | ***** | ***** | ****** |  | ***** | ***** | ***** |
| Eichler et al 2016 | ***** | ***** | ***** | ***** | ****** |  | ***** | ***** |  |
| van Bastelaar et al 2016 | ***** | ***** | ***** | ***** | ***** |  | ***** |  |  |
| van Bastelaar et al 2017 | ***** | ***** | ***** | ***** | ****** |  | ***** | ***** |  |
| Ouldamer et al 2015 | ***** | ***** | ***** | ***** | ***** |  | ***** | ***** |  |
| Khater et al 2015 | ***** | ***** | ***** | ***** | ***** |  | ***** | ***** | ***** |
