# Supplementary material for: Flap fixation in preventing seroma formation after mastectomy: an updated meta-analysis
Source: Updates Surg. 2021 Apr 22;73(4):1307–14. doi: 10.1007/s13304-021-01049-9 (PMC8397649; doi:10.1007/s13304-021-01049-9)
Supplement: Supplementary file 3 — Supplementary file3 (DOCX 18 KB) Appendix 2: PRISMA flowchart [file 13304_2021_1049_MOESM3_ESM.docx]

Records excluded with reason (n = 40)

For title/abstract (n = 32)

For language (n = 5)

No Full Text Avaible (n = 3)

Full-text articles excluded, with reasons
(n = 4)

Review/Animal model/Case report (n = 3)

Lack of data (n = 1)

Additional records identified through other sources
(n = 0)

Records identified through database searching
(n = 56)

## Included

## Eligibility

Full-text articles assessed for eligibility
(n = 16)

Studies included in quantitative synthesis (meta-analysis)
(n = 12)

Studies included in qualitative synthesis
(n = 12)

Records screened
(n = 56)

Records after duplicates removed
(n = 56)

## Identification

## Screening
